# Strong and Elastic Chitosan/Silk Fibroin Hydrogels Incorporated with Growth-Factor-Loaded Microspheres for Cartilage Tissue Engineering

**DOI:** 10.3390/biomimetics7020041

**Published:** 2022-04-07

**Authors:** Qing Min, Danlei Tian, Yuchen Zhang, Congcong Wang, Ying Wan, Jiliang Wu

**Affiliations:** 1 School of Pharmacy, Hubei University of Science and Technology, Xianning 437100, China; baimin0628@hbust.edu.cn (Q.M.); zhangych@hbust.edu.cn (Y.Z.); 2 College of Life Science and Technology, Huazhong University of Science and Technology, Wuhan 430074, China; tiandanlei@hust.edu.cn (D.T.); congcongwang@hust.edu.cn (C.W.)

**Keywords:** composite hydrogel, core-shell microspheres, strength and elasticity, kartogenin, platelet-derived growth factor BB, cartilage tissue engineering

## Abstract

An emulsification method was developed for fabricating core-shell microspheres with a thick shell layer. Kartogenin (KGN) and platelet-derived growth factor BB (PDGF-BB) were respectively loaded into the core portion and the shell layer of the microspheres with high loading efficiency. The optimally built microspheres were combined with chitosan (CH) and silk fibroin (SF) to construct a new type of composite hydrogel with enhanced strength and elasticity, using genipin or/and tyrosinase as crosslinkers for the intended use in cartilage tissue engineering. The composite hydrogels were found to be thermo-responsive at physiological temperature and pH with well-defined injectability. Rheological measurements revealed that they had an elastic modulus higher than 6 kPa with a high ratio of elastic modulus to viscous modulus, indicative of their mechanically strong features. Compressive measurements demonstrated that they possessed well-defined elasticity. In addition, some gels had the ability to administer the temporal separation release of PDGF-BB and KGN in an approximately linear manner for several weeks. The released PDGF-BB was found to be bioactive based on its effects on Balb/c 3T3 cells. The composite gels supported the growth of seeded chondrocytes while preserving their phenotype. The results suggest that these composite gels have the potential for endogenous cartilage repair.

## 1. Introduction

Articular cartilage is a specific type of tissue that connects to the ends of bones in synovial joints and endows the joint with several vital biomechanical functionalities, such as wear resistance, load-bearing capacity, and shock absorption [1]. Adult articular cartilage is known to have very limited capacity to self-repair once injured or pathogenically degraded, due to its avascular features [2]. Several kinds of surgical techniques have typically been used for articular cartilage repair, mainly including arthroscopic lavage with or without corticosteroids, microfracture, subchondral bone drilling, abrasion arthroplasty, and mosaicplasty. However, outcomes associated with these treatments frequently remain unpredictable, and many clinical cases have not proven to be successful from a long-term perspective [2]. Numerous efforts have thus been made to search for alternative therapies. Nowadays, tissue engineering, which commonly involves the combination of cells, biomaterials, and signaling molecules, has emerged as a new strategy for articular cartilage repair and regeneration [2,3].

Among the available biomaterials used for cartilage tissue engineering, injectable polymer hydrogels have received a lot of interest because they can form into complex shapes in good alignment with the morphology of irregular defects in a minimally invasive manner [4]. Chitosan (CH) is a natural polysaccharide and has been extensively investigated for hydrogel applications due to its advantageous properties [5]. A type of glycerophosphate (GP)-crosslinked CH hydrogel has been thoroughly investigated for different biomedical applications because of its safe ingredients, ease of preparation, and thermo-responsiveness at physiological temperature and pH [6]. Despite its wide-ranging usability, the CH/GP gel has very low strength and is mechanically fragile with a fast in vivo degradation rate; this intrinsically limits its utilization in cartilage repair and regeneration, where the applied gels should be mechanically strong and elastic [1,2,3,4].

Silk fibroin (SF) is another kind of natural polymer which has been widely used in tissue engineering due to its various advantages, such as low inflammation response, promotion of cell adhesion, facilitation of three-dimensional colonization for many types of cells, robust mechanical properties, and well-defined degradation tolerance [7]. SF can be processed into hydrogels via several different physical or chemical methods [8]. Genipin is a kind of naturally sourced molecule with notably better biocompatibility compared to many chemical crosslinkers. It has been used as a crosslinker for the preparation of CH and SF hydrogels under mild conditions [9,10]. Nevertheless, the amount of genipin used in CH and SF hydrogels must be lower than certain thresholds to ensure the in vivo safety of the resulting gels. As a result, genipin-crosslinked CH and SF hydrogels have limited enchantment. In addition, genipin-involved crosslinking reactions are time-consuming, and the gelation time for genipin-crosslinked CH and SF hydrogels is long [9,10]. Thus, genipin alone is not suitable for in situ preparation of CH or SF hydrogels.

Tyrosinase (TYR), a polyphenol oxidase, is a more biocompatible biomolecule than genipin. TYR can oxidize the tyrosine into *o*-Quinone, and the latter is able to react with the amino group through the Michael addition reaction [11]. Accordingly, TYR should be able to catalyze the crosslinking reaction between SF and CH by oxidizing the tyrosine residues of SF. Many studies have suggested that a polymer hydrogel with multiple networks could be substantially enhanced in terms of its stability, mechanical performance, and degradation tolerance when compared to a single network gel [4,12,13,14]. Taking into account the merits of TYR and genipin, mechanically strong CH/SF composite hydrogels with multiple networks could be developed, provided that genipin and TYR are conjointly employed in an effective manner. Considering that the reaction rate of genipin- or TYR-involved crosslinking is slow, it may be practicable to use GP together with genipin and TYR for the preparation of composite gels since GP can gelatinize a CH solution within a reasonable period of time [6].

In addition to the employment of desirable hydrogels for housing cells, the local application of bioactive molecules is another effective method for cartilage repair and regeneration [15]. Some active molecules, mainly including cytokines and growth factors, serve multiple functions and are responsible for the individual or combined response behaviors in the migration, proliferation, and differentiation of affected cells; these molecules can play complex roles throughout different stages of the whole repair process [16]. Among the multifunctional growth factors, platelet-derived growth factor BB (PDGF-BB) performs several key functions in stimulating cell chemotaxis, mitogenesis, and hematopoiesis; these functions have been commonly utilized alone or in combination for the repair of cartilage lesions, bone defects, and wound healing [17,18,19]. The chemotactic capacity of PDGF-BB for recruiting cells from mesenchymal sources has positioned PDGF-BB as an important factor for applications relating to the endogenous repair of cartilage or bone defects [18,20]. The local administration of PDGF-BB can enhance tissue repair by inducing the recruitment of mesenchymal stem cells (MSCs) from the host tissue surrounding the cartilage defect, and facilitating their proliferation in situ [18,19,20]. For the repair of full-thickness cartilage defects, it is feasible to apply an appropriate amount of PDGF-BB at the defect site to recruit MSCs in situ. In this way, the participation of exogenous seed cells is not required. Despite available chemotaxis from PDGF-BB, in the endogenous repair mode, how to promote chondrogenic differentiation of the recruited MSCs is another important issue that needs addressing.

Kartogenin (KGN), a synthetic heterocyclic molecule, has been demonstrated to promote the differentiation of primary human MSCs into cartilage nodules by mediation through the CBFβ-RUNX1 signaling pathway [21]. Importantly, the nodules produced had markers specific to cartilage, such as type II collagen and aggrecan and, at the same time, did not have markers for hypertrophic chondrocytes or bone cells, confirming KGN’s capacity for specifically inducing chondrogenic differentiation of MSCs [22]. In addition, the results, based on long-term culture of differentiated cells with KGN, revealed that the levels of type II collagen, aggrecan and metalloproteinase inhibitor increased, signifying that KGN facilitates maintenance of the cartilage phenotype and prevention of extracellular matrix damage caused by metalloproteinase [21,22,23]. Accordingly, KGN should be a suitable candidate to work with PDGF-BB to stimulate the chondrogenic differentiation of recruited MSCs, and to sustainably maintain the cartilage phenotype of the differentiated cells.

In this study, an effort was made to develop a new type of composite hydrogel with strong and elastic characteristics while having the ability to administer the release of PDGF-BB and KGN. Core-shell microspheres (MPs), with a thick shell layer, were first built to load KGN and PDGF-BB using alginate (ALG) and CH as core and shell materials, respectively, while employing GP and ethylene glycol diglycidyl ether (EGDE) as crosslinkers. The optimally generated MPs were embedded into CH and SF hydrogel to construct composite gels through genipin or/and tyrosinase crosslinking. Composite gels were found to have the ability to support the growth of seeded chondrocytes and matrix deposition, control the release of PDGF-BB and KGN and preserve the bioactivity of the released PDGF-BB, suggesting their potential to function as an endogenous repair material for cartilage tissue engineering.

## 2. Results

### 2.1. Parameters of Microspheres

High EE is one of the key issues that need to be considered for the appropriate use of PDGF-BB due to the high cost of PDGF-BB. BSA was thus used as a substitute for PDGF-BB to optimize the compositional and processing parameters to achieve desired core-shell MPs with a thick shell layer, sufficient DL and high EE. Several major parameters were optimized with the orthogonal design method; the optimized parameters are shown in the experimental section.

In the preparation of PDGF/KGN-M MPs, BSA was also employed as a complementary component and the applied BSA quantity was set to be the same as that used for the preparation of BSA/KGN-M MPs. By doing so, BSA can assist in effectively encapsulating PDGF-BB inside the shell layer of PDGF/KGN-M MPs via physical interactions due to their difference in pI values, as shown in the experimental section. Blank core-shell MPs without any cargo load, denoted BLANK-M MPs, were also prepared and used for subsequent characterization and evaluation of the hydrogels. Three kinds of MP were prepared under the same processing conditions, and some of their parameters are summarized in Table 1.

**Figure 1:**
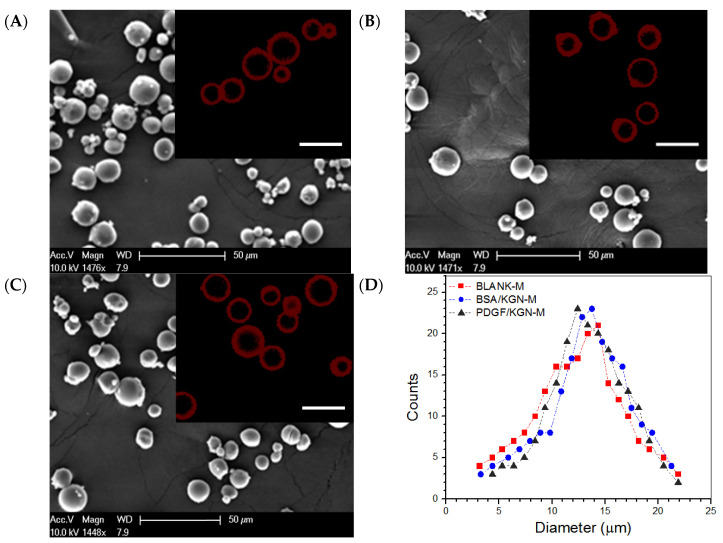
SEM images for BLANK-M MPs (**A**), BSA/KGN-M MPs (**B**) and PDGF/KGN-M MPs (**C**) (inserted fluorescence images denote the shell layer of MPs, scale bar: 20 μm); and size distribution (**D**) for three kinds of MPs.

Several representative micrographs for different kinds of MPs are presented in Figure 1A–C. These MPs were seen to be similar in morphology and had quite varied sizes ranging from a few microns to around 25 microns. The inserted fluorescence images show that they had a well-constructed core-shell structure. The curves in Figure 1D indicate that these MPs had similar size distributions and each had an approximately symmetrical distribution. The average diameter and the average thickness of the shell layer for three kinds of MPs were also similar without significant differences (Table 1), suggesting that BLANK-M MPs can serve as an equivalent analog to BSA/KGN-M or PDGF/KGN-M MPs in the subsequent preparation and characterization of composite gels. Data shown in Table 1 indicate that BSA/KGN-M MPs had high EE for both BSA and KGN, and in the case of PDGF/KGN-M MPs, the EE for PDGF-BB reached about 87%. The results in Figure 1 and Table 1 demonstrate that core-shell MPs with a thick shell layer and a high EE for both PDGF-BB and KGN were successfully built via the developed emulsification method.

### 2.2. Major Parameters for Gels Embedded with BLANK-M Microspheres

BLANK-M MPs were embedded into the gel system composed of CH and SF at an optimized ratio of CH to SF to build several kinds of composite gels while crosslinking these gels using genipin or/and TYR. The formulations for the composite gels are illustrated in Table 2. Typical photos obtained from inverted tube tests for the sol-gel transition of several gels are displayed in Figure S1. These composite solutions underwent clear phase transition after being incubated at 37 °C for various periods, confirming that they had gelable features at physiological temperature. In comparison to GL-1 gel, GL-3, GL-5 and GL-7 gels had much shorter gelation times. Considering that the reaction rate of genipin- or TYR-involved crosslinking is slow, the shortened gel times of GL-3, GL-5 and GL-7 gels can be attributed to the fact that BLANK-M MPs in these gels significantly increased the concentration of the gels and, thus, promoted a faster gel process.

**Figure 2:**
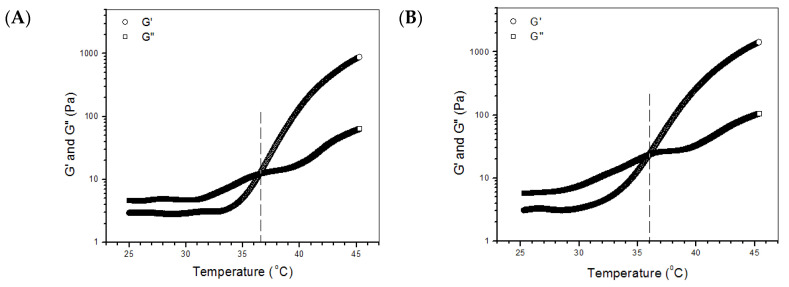

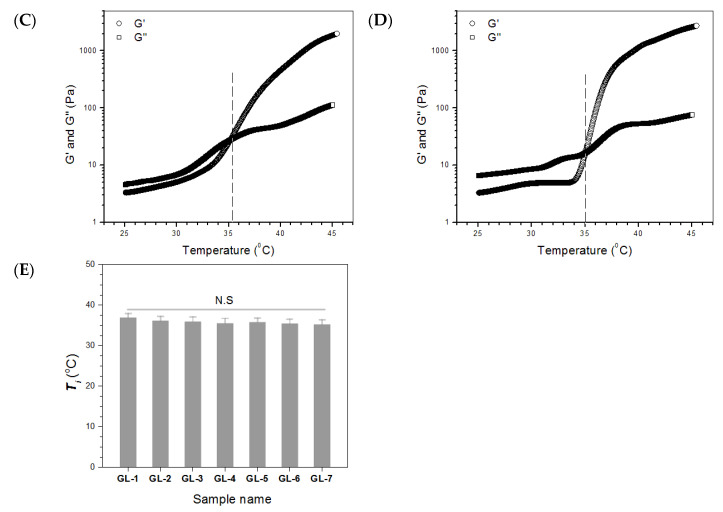
Representative temperature-dependence functions of G′ and G′′ for GL-1 (**A**), GL-3 (**B**), GL-5 (**C**) and GL-7 gels (**D**); and incipient gelation temperature (T_i_) for all gels (**E**) (see Table 2 for their parameters), N.S., not significant.

Several temperature-dependence curves of G′ and G′′ for GL-1, GL-3, GL-5 and GL-7 gels are presented in Figure 2. GL-1 gel had a T_i_ of around 36.7 °C, and T_i_ for GL-3, GL-5 and GL-7 gels was a little lower. The samples in Table 2 were all tested for their T_i_ and data obtained are shown in Figure 2E. The bar graphs show that there were no significant differences in T_i_ for these gels, indicating that the addition of BLANK-M MPs, and the presence of genipin or/and TYR, had insignificant effects on T_i_ of the gels. Data in Table 2 indicate that pH for all gels was around 7 without significant differences, and that, compared to GL-1, other gels had a much shorter gelation time. Results in Figures S1 and S2 and Table 2 indicate that the composite solutions shown in Table 2 had thermo-sensitive features at near-physiological temperature and pH, and that the resulting gels had a rational gelation time.

**Figure 3:**
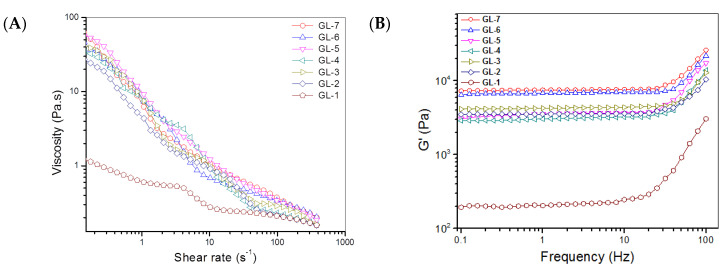

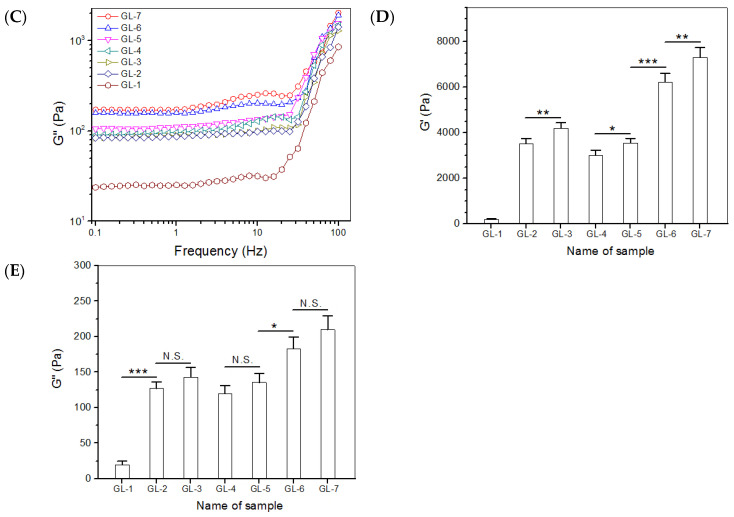
Shear-rate dependent variations of viscosity ((**A**), 25 °C), frequency-dependent changes of G′ ((**B**), 37 °C) and G′′ ((**C**), 37 °C), and average value of G′ ((**D**), 37 °C) and G′′ ((**E**), 37 °C) at 1 Hz for different gels (***, *p* < 0.001; **, *p* < 0.01; *, *p* < 0.05; N.S., not significant).

The gels in Table 2 were further measured for their viscosity variation against shear rates at 25 °C to evaluate their injectability, and the obtained data are plotted in Figure 3. GL-1 had very low viscosity over the shear-rate measurement range. GL-*i* (*i* =2, 3, 4, 5, 6 and 7) gels were significantly more viscous compared to GL-1. Nevertheless, they all had shear-thinning properties, with incipient viscosity ranging between approximately 30 and 60 Pa.s at very low shear-rate, and gradually decreasing viscosity, falling below 2.0 Pa.s when the shear rate was 10 s^−1^ or higher. Considering that the injection of composite solutions is usually conducted at room temperature, the results in Figure 3A demonstrate their well-defined injectability.

Frequency-dependence spectra of G′ and G′′ at 37 °C for gels in Table 2 were measured; and the results are also presented in Figure 3. G′ of these gels was proximately linear in the frequency interval with an upper limit of around 10 Hz. All these gels had much lower G′′ when compared to their respective G′ values. Several specimens for each gel were measured to calculate average G′ and G′′ at 1 Hz; and relevant data are graphed in Figure 3D,E. GL-1 gel had a G′ of about 200 Pa; the G′ values for GL-2, GL-3, GL-4 and GL-5 gels were higher than 3 kPa; notably, GL-6 and GL-7 gels showed G′ higher than 6 kPa, being much greater than the G′ values for the GL-2, GL-3, GL-4 and GL-5 gels. In general, a mechanically strong hydrogel has a large G′ and, at the same time, the ratio G′ to G′′ should be significantly greater than 10 [24]. Based on data shown in Figure 3D,E, the G′/G′′ ratio for GL-*i* (*i* = 1, 2, 3, 4, 5, 6 and 7) gels were estimated to be around 9.8, 27.7, 29.5, 25.2, 26.3, 33.9 and 34.9, respectively. Among these, the GL-2, GL-3, GL-4, GL-5, GL-6 and GL-7 gels behaved like mechanically strong gels.

### 2.3. Compressive Mechanical Evaluation

**Figure 4:**
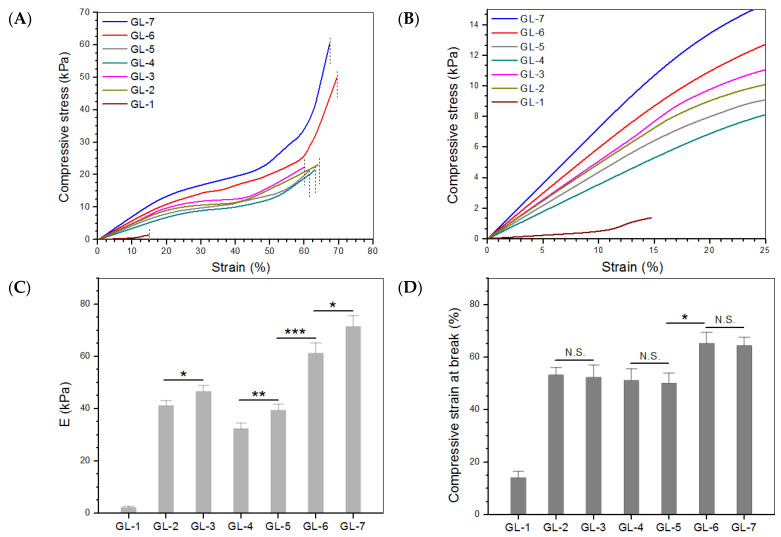
Representative strain-stress curves (**A**), dash lines denote the compressive strain at the break), enlarged view of strain-stress curves in low strain region (**B**), average compressive modulus (**C**) and compressive strain at the break (**D**) for different gels (***, *p* < 0.001; **, *p* < 0.01; *, *p* < 0.05; N.S., no significance).

Representative stress-strain curves for the gels illustrated in Table 2 are presented in Figure 4A and the enlarged view of these curves in the low strain region is displayed in Figure 4B. The calculated compressive modulus (E) and strain at the break based on more samples are exhibited in Figure 4C,D. The GL-1 gel had very low compressive stress at break, whereas its strain at the break was estimated to be around 14%, symbolizing its weak and fragile characteristics. The results in Figure 3 and Figure 4 confirm the inapplicability of GL-1 gel in cartilage repair. In contrast to GL-1, GL-*i* (*i* = 2, 3, 4, 5, 6 and 7) gels can be considered as strong elastomers. There were three identifiable phases in the strain-stress curves for GL-*i* (*i* = 2, 3, 4, 5, 6 and 7) gels: rapid stress rise at small strain, slow stress rise over a plateau-like strain region, and further relatively faster stress rise at large strain. In particular, the stress for GL-6 and GL-7 gels sharply increased before reaching their compressive break strains, signifying that they behaved as strong elastomers. The bar graphs in Figure 4C,D show that the combined use of genipin and TYR to crosslink the gels greatly enhanced their strength while significantly improving their elasticity compared to the use of genipin or TYR alone, and that the content of MPs also contributed to significant enhancement of the gels.

### 2.4. Cell Growth and Matrix Deposition

**Figure 5:**
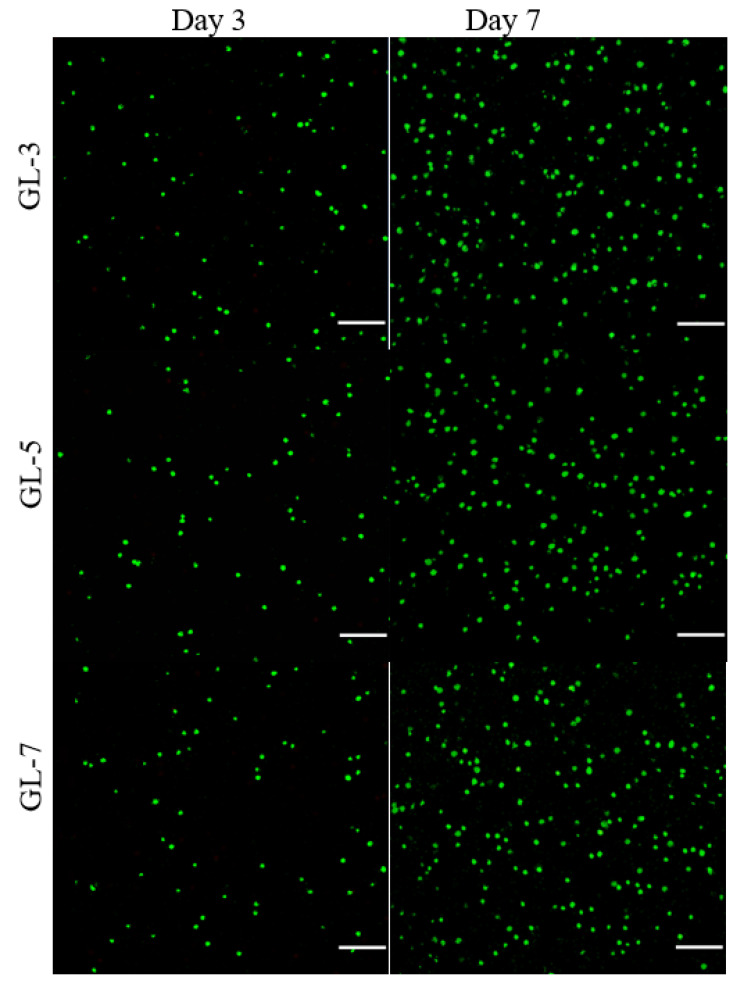
Confocal images for stained chondrocyte cells (green: viable cells; red: dead cells; scale bar: 100 μm).

GL-3, GL-5 and GL-7 gels had similar compositions except for the differences in the applied crosslinkers, and thus, they were selected for cell culture. Figure 5 shows several fluorescence images for the stained chondrocytes that were seeded in gels and cultured for various periods. Very few dead cells were detected from these gels after cell-gel constructs were cultured for 3 or 7 days, and cell density in the images corresponding to 7-day culture was notably higher than that corresponding to 3-day culture. These images suggest that the seeded chondrocytes had high viability, and the gels had similar ability to support their growth.

**Figure 6:**
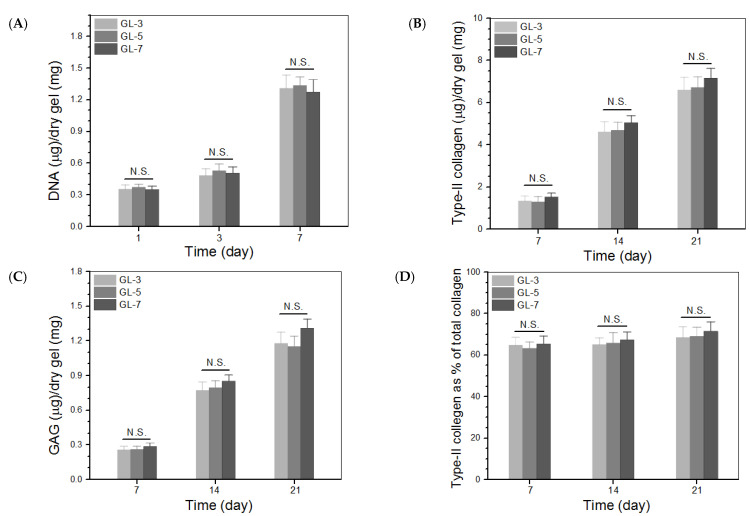
DNA amounts detected from cell-gel constructs (**A**), measured amounts of type II collagen and glycosaminoglycan (GAG) in gels (**B**,**C**), and percentage of type II collagen in total collagen for gels (**D**) (N.S., not significant).

Figure 6A delineates results for the cell proliferation in different gel matrices. Cell growth broadly underwent two phases: fewer cells grew from day 1 to day 3, and cells grew faster from day 3 to day 7. The slow cell growth in the first phase can be ascribed to cell attachment with subsequent population recovery, and the second phase can be attributed to the occurrence of cell proliferation. There were no significant differences in detected DNA quantities among these gel groups during one-week culture, suggesting that they had similar ability to support proliferation of seeded cells. Considering the differences in the use of crosslinkers among GL-3, GL-5 and GL-7 gels, the results in Figure 5 and Figure 6 indicate that the applied amounts of genipin or/and TYR were within a safe range.

Type-II collagen, glycosaminoglycan (GAG) and aggrecan are typical marker molecules in the extracellular matrix (ECM) of articular cartilage [1]. Amounts of type-II collagen and GAG in cell-seeded gels were therefore determined; the data obtained are plotted in Figure 6B,C. The bar graphs in Figure 6B show that the amount of type-II collagen deposited markedly increased as the culture time advanced, and that there were no significant differences in the amount of type-II collagen detected from these gels. In the situation of GAG deposition, a similar upward trend in comparison to the type-II collagen deposition was also observed. The ratio of type-II collagen to total collagen can be used to assess whether the chondrocytes seeded in hydrogels can predominantly synthesize their specific matrix component, type-II collagen, which enables determination of whether the seeded chondrocytes are able to maintain their phenotype [25,26]. The data presented in Figure 6D show that the proportion of type-II collagen to total collagen reached a level higher than 60% from day 7 to day 21 without significant differences among these gels, suggesting that the collagen amount in ECM predominantly belonged to type-II collagen. It can thus be inferred that the function and phenotype of the seeded chondrocytes was effectively preserved.

### 2.5. In Vitro Release

**Figure 7:**
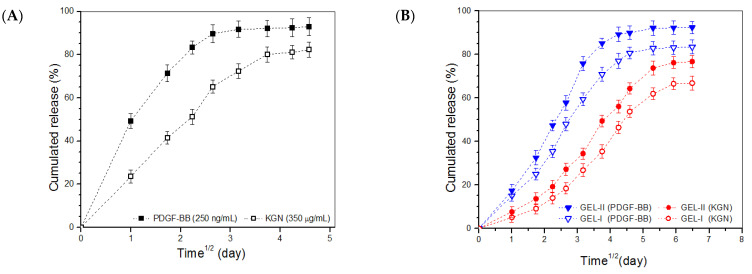

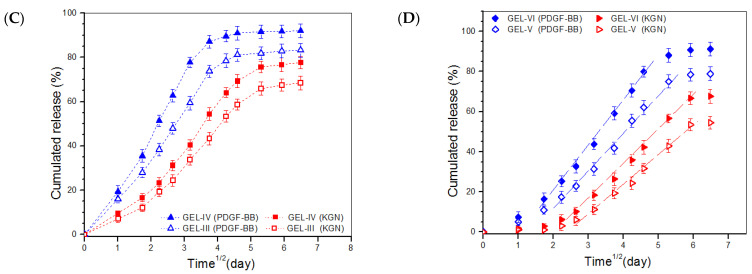
Cumulative release profiles for PDGF/KGN-M MPs (**A**) and composite gels containing varied amounts of PDGF-BB and KGN (**B**–**D**) (see Table 1 and Table 3 for their respective parameters).

PDGF/KGN-M MPs were used to replace BLANK-M MPs for the preparation of growth factor-loaded composite hydrogels following the formulations presented in Table 2; the major parameters for the gels are provided in Table 3. PDGF/KGN-M MPs were first detected to assess their release behavior; and their release patterns are represented in Figure 7A. PDGF/KGN-M MPs released about 50% of the incorporated PDGF-BB and around 23% of the loaded KGN on the first day, and the two curves reached to their respective plateau regions at day 7 and day 14, respectively. Figure 7A confirms that PDGF/KGN-M MPs had very limited ability to administer the release of PDGF-BB and KGN because of their severe initial burst release characteristics and the fast release rates for both PDGF-BB and KGN. Figure 5B,C show the release profiles for the GEL-I, GEL-II, GEL-III and GEL-IV gels. These curves illustrate that the burst release from these gels was greatly suppressed and the release rates of PDGF-BB and KGN were also significantly slowed down compared to PDGF/KGN-M MPs. In addition, it can be observed that the release rates of PDGF-BB and KGN were significantly regulated by the amount of PDGF/KGN-M MPs applied. In Figure 7D, the release patterns for GEL-V and GEL-VI reveal that both PDGF-BB and KGN were released in an approximately linear fashion longer than two weeks for the former and three weeks for the latter. It is worth pointing out that the initial loads of PDGF-BB and KGN in PDGF/KGN-M MPs had already been pre-specified taking into account the incomplete release from these formulated gels, and that the released amounts of PDGF-BB and KGN from the gels were sufficient for their in vivo application [27,28,29,30].

Despite the complexity of release systems, several empirical models have been developed to describe the kinetics of some release systems [31]. In the case of swellable polymer matrices, the kinetic behavior of drug release can be estimated from the following semi-empirical equation [31,32]:

*M*_t_/*M*_∞_ = *kt^n^* or log[*M*_t_/*M*_∞_] = log*k* + *n*log*t* (1)

where *M*_t_/*M*_∞_ is the fractional release of the drug at time *t*, *k* is a constant correlated to the structural and geometric characteristics of release system, as well as the release rate, and *n* is the release exponent, indicative of the drug release mechanism. Based on equation (1), *n* and *k* for different release systems were calculated and relevant data are summarized in Table 4.

Generally speaking, an *n* value below 0.45 is indicative of a Fickian diffusion mechanism, an *n* value ranging between 0.45 and 0.85 indicates anomalous transport controlled by both diffusion and swelling, and an *n* value larger than 0.85 signifies super case-II transport which is related to polymer relaxation during swelling [31,32,33]. Data in Table 4 indicate that the *n*-values of PDGF/KGN-M MPs were less than 0.45, suggesting that release of PDGF-BB or KNG from these MPs involved Fickian diffusion. All tested gels had *n* values ranging between 0.55 and 0.99, indicating that the release of PDGF-BB or KNG from these gels followed an anomalous transport mechanism related to Fickian diffusion and case-II transport [31,32,33,34,35].

The bioactivity of the released PDGF-BB was evaluated by testing its chemotactic capacity on fibroblasts [36]. GEL-II, GEL-IV and GEL-VI gels were selected for the bioactivity evaluation in consideration of their higher initial PDGF-BB load when compared to the other three kinds of gels. Based on the Transwell culture method described in the experimental section, the number of migrating Balb/c 3T3 cells corresponding to GEL-II, GEL-IV and GEL-VI gel groups was graphed in Figure S2. Bar graphs showed a significant dose-dependency trend for PDGF-BB stimulated cell migration in each group, with the number of migrating cells greatly increasing by more than 6.5 times when the amount of PDGF-BB increased from 1 to 10 ng/mL. Importantly, there were no significant differences in the number of migrating cells among the gel groups and the free PDGF-BB group at all applied PDGF-BB dose levels, confirming that the released PDGF-BB and free PDGF-BB were almost equally effective, and that the bioactivity of the loaded PDGF-BB in these composite gels had been well preserved.

## 3. Discussion

ALG has been extensively investigated for biomedical applications in the form of MPs because it is biodegradable, has good in vivo safety, and can be conveniently crosslinked by some divalent ions, such as Ca^2+^, Sr^2+^ and Zn^2+^ [37]. CH is another kind of biodegradable polysaccharide which has been widely used for various biomedical purposes owing to its many advantages [5,9]. In light of the cationic features of CH, CH can interact with anionic ALG to build polyionic hydrocolloids [38,39] which are conducive to the construction of layered ALG/CH composites where an appropriate processing technique is employed. Hence, ALG and CH were selected as the core and shell materials for building core-shell MPs, respectively. Polymer-based core-shell MPs can be fabricated via different processing techniques. One of the common techniques is first to produce a kind of MP, and then to use these as the core to fabricate core-shell MPs by surface coating. However, the thickness of the coating layer is generally very thin relative to the diameter of such built MPs due to the low concentration of applicable coating solutions [40]. The thin coating layer of MPs prepared in this way is usually left empty and serves only as a barrier to delay the release of the cargo loaded inside their core portion [41,42]. Another commonly used technique for the preparation of core-shell MPs is the multiple emulsion method [43]. Despite common use, the preparation of core-shell MPs with a thick shell layer via the multiple emulsion method is often a tough test of the researcher’s experience and patience because the preparation of MPs via this technique is affected by a variety of factors such as materials employed, the emulsion system, the processing method and processing conditions, and so on. In particular, when such prepared core-shell MPs are intended for use in the delivery of protein molecules, how to preserve the activity of loaded protein molecules during the preparation of MPs is an important issue that needs to be pre-devised.

In the present study, efforts were made to prepare core-shell MPs with a thick shell layer and small size with the selected CH while employing optimal processing parameters. As described in the experimental section, two kinds of crosslinkers, GP and EGDE, were sequentially utilized to solidify the shell layer of BSA/KGN-M MPs. GP was able to rapidly gelatinize CH molecules to quickly block BSA inside the shell layer, and additionally, did not interfere with the structure and functioning of BSA because the CH chains were bound via GP-associated ionic linkages, which was particularly important for subsequent PDGF-BB encapsulation. After preparatory solidification by GP, it was difficult for EGDE to penetrate the shell layer, and thus, EGDE could only crosslink the surface of the shell layer due to the very small amount of EGDE used. EGDE-involved crosslinking played a role in preventing BSA loss during the preparation and post-processing of MPs, and slowing down the release of cargoes. Based on the orthogonal design and numerous trials, the compositional proportion and processing parameters for BSA/KGN-M MPs were optimized to endow the MPs with appropriate sizes suitable for injection and high EE for both BSA and KGN.

With respect to the preparation of PDGF/KGN-M MPs, the protection of PDGF-BB activity was pre-considered in the design and preparation of the core-shell MPs. Since the effective PDGF-BB dose required for in vivo cartilage repair is very low [44,45], a small amount of BSA was employed to provide physical protection of PDGF-BB activity via ionic interactions between BSA and PDGF-BB, while preventing PDGF-BB loss during the preparation of PDGF/KGN-M MPs.

The CH/GP gel forms by GP-involved physical crosslinking and is mechanically weak and fragile (Figure 3 and Figure 4). Introducing additional ingredients into the gel system through physical mixing could enhance the strength of resulting composite gels to a certain extent, but the elasticity of the gels is usually not effectively improved [42,43,44]. Other concerns that could arise from physical mixing are that the resulting composite gels may lose their injectability or thermo-responsive capacity at physiological temperature and pH due to the introduced ingredients. In the present instance, optimized amounts of SF and MPs were introduced into the CH/GP gel system while employing chemical crosslinking using genipin or TYR alone or in combination. The composite gels, crosslinked by a single crosslinker or both, not only maintained their injectability and sol-gel transition well under physiological conditions, but they also showed greatly enhanced strength and elasticity (Figure 2, Figure 3 and Figure 4 and Table 2). Remarkably, the gels prepared with both genipin and TYR, namely, GL-6 and GL-7, had much higher compressive moduli and stresses at the break when compared to gels prepared with either genipin or TYR alone (Figure 4), providing evidence for the occurrence of multiple crosslinking inside GL-6 and GL-7 gels. Moreover, these composite gels were also endowed with strong capability for supporting the growth and matrix deposition of seeded chondrocytes due to the use of optimized amounts of genipin or/and TYR, as evidenced by Figure 5 and Figure 6.

The basic ingredients for successful endogenous articular cartilage repair are cells, and the appropriate stimuli to guide their growth and ECM synthesis towards building hyaline articular cartilage [1,2,12,46,47,48]. Endogenous cartilage repair that relies on the recruitment of chondrocytes is usually insufficient due to the low metabolic activity and very small population of chondrocytes; and hence, MSCs and some progenitor cells have been considered as better choices for endogenous cartilage repair [48,49,50]. Recruiting stem cells and facilitating their migration to the defect site could be achieved by using biological or physicochemical cues provided by the employed materials and bioactive molecules. In the case of full-thickness articular cartilage repair, the combined use of PDGF-BB and KGN would be a feasible strategy for recruiting MSCs from bone marrow or synovium by taking advantage of PDGF-BB and for inducing their subsequent chondrogenic differentiation by KGN stimulus. The presently developed composite gels showed ability to control the release of PDGF-BB and KGN in a temporal separated manner at varied release rates (Figure 7). The sustainable and controlled release of PDGF-BB and KGN can be primarily ascribed to the contribution of the gel matrix. In composite gels, CH and SF molecules form a capping layer on the surface of PDGF/KGN-M MPs and the gel network also resists the diffusion of PDGF-BB and KGN, both reducing the release rate of PDGF-BB and KGN. GEL-V and GEL-VI gels were multiply crosslinked by genipin and TYR and, thus, they would be predicted to have a much higher crosslinking density compared to other gels crosslinked by genipin and TYR alone. Accordingly, they possessed enhanced ability to slow down the release of both PDGF-BB and KGN, resulting in approximately linear release patterns (Figure 7D).

The effective bioactivity maintenance of PDGF-BB can be attributed to the joint contributions of MPs and gel matrices. In this study, the protection of PDGF-BB activity was pre-considered in the design and preparation of the core-shell MPs. Since the required PDGF-BB dose for in vivo cartilage repair is very low [27,28], a small amount of BSA was employed as a complementary component to provide physical protection to PDGF-BB. In addition, GP was used to gelatinize the CH shell layer of MPs via physical crosslinking before the use of EGDE, further contributing to physical protection of PDGF-BB. With respect to the preparation of composite gels, the content of genipin and TYR was optimized within a safe range. As a result, the bioactivity of PDGF-BB released from these gels was well preserved.

The presently devised composite gels have strong and elastic characteristics with thermo-responsive and injectable properties. They are able to support the growth and matrix synthesis of chondrocytes while maintaining their phenotype. These gels also have ability to administer the release of PDGF-BB and KGN in a controlled manner. These results suggest that the optimally achieved gels have potential to function as an endogenous repair material for cartilage repair via the synergistic effects derived from PDGF-BB, KGN and the gel matrix. Further studies on the degradation tolerance of the composite gels and their performance for endogenous articular cartilage repair are now underway, and relevant results will be presented in separate reports.

## 4. Materials and Methods

### 4.1. Materials

Chitosan (CH, viscosity: 100-200 mPa·s), sodium alginate (ALG, 180–220 mPa⋅s), GP, KGN, genipin, EGDE and bovine serum albumin (BSA, isoelectric point (pI): ca.4.8) were supplied by Aladdin Inc. (Shanghai, China). CH was treated in a 50 wt% NaOH aqueous solution to increase its deacetylation degree to around 95.3% using the method described in our previous study [51]. The PDGF-BB (pI: ca.9.8) and the PDGF-BB ELISA kit were bought from R&D Systems (Minneapolis, MN, USA) and Invitrogen (Waltham, MA, USA), respectively. Tyrosinase (EC 1.14.18.1) (≥1000 units/mg, T-3824) was purchased from Sigma-Aldrich (Shanghai, China). Other reagents and chemicals were of analytic grade and obtained from Sinopharm, China (Shanghai, China).

SF was isolated from cocoons using the method described in our previous study [52]. The obtained SF solution was diluted to 1.0 wt% of final concentration with storage at 4°C for further use. Rhodamine B (RDB) was conjugated onto CH to synthesize some conjugates (RDB-CH) following a reported method [39]. The selected RDB-CH was used together with CH to image the shell layer of some MPs.

### 4.2. Preparation of Core-Shell Microspheres with Load of KGN and PDGF-BB

BSA was first used as a substitute for PDGF-BB to prepare core-shell MPs to optimize the preparation of MPs and the load efficiency to save costly PDGF-BB. A typical preparation process was as follows: KGN was dissolved in dimethyl sulfoxide (DMSO) and this KGN solution was added dropwise to a 2.0% aqueous ALG solution with stirring to reach a KGN/ALG ratio of 100 μg KGN/mg ALG. This mixture was assigned for the preparation of the core portion of MPs. A 3.0% CH solution was prepared by dissolving CH in a 0.5% aqueous acetic acid solution and was added dropwise with a 0.135% BSA solution in PBS to reach a BSA/CH ratio of 5 μg BSA per mg CH. The BSA/CH mixture was allocated for building the shell layer of MPs.

To an oil phase consisting of a mixture of liquid paraffin/vacuum pump oil (1:1) and Span-80 (5%), the KGN/ALG mixture was slowly introduced within 1 h using a syringe while vigorously stirring (10,000 rpm) with a high-speed homogenizer to generate a W/O emulsion. An aqueous CaCl_2_ solution was then slowly dropped into the emulsion with stirring for 10 min to slightly solidify ALG. After that, the BSA/CH mixture was added dropwise to the emulsion within 1 h with stirring. Subsequently, the emulsion system was placed in a water bath maintained at 37 °C, and an aqueous GP solution was added to the emulsion with stirring for 30 min to solidify the CH shell layer, followed by the addition of aqueous EGDE solution for the further solidification of the CH shell layer for 2 h. Thereafter, core-shell MPs were retrieved by centrifugation, washed with petroleum ether, ethanol and water in order, and lyophilized. Several key parameters were optimized: final oil/water volume ratio, 6:1; CaCl_2_/ALG weight ratio, 3:1; GP/CH weight ratio, 2.8:1; and EGDE/CH weight ratio, 0.02:1. The MPs produced with optimal parameters were referred to as BSA/KGN-M MPs.

**Figure 8:**
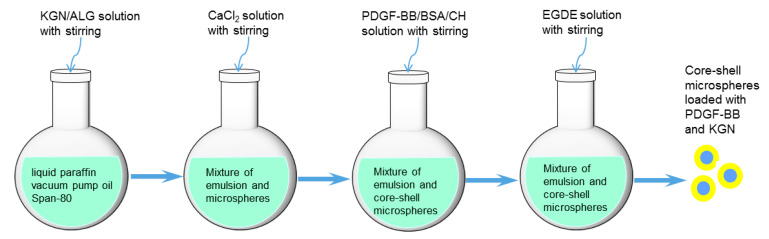
Schematic illustration for preparation of core-shell microspheres loaded with PDGF-BB and KGN.

The MPs loaded with PDGF-BB and KGN (denoted PDGF/KGN-M MPs) were prepared using the same protocol applied for the preparation of BSA/KGN-M MPs, except that PDGF-BB was added to the BSA solution before the BSA solution was added to the 3.0% CH solution. A schematic illustration for the preparation of the core-shell microspheres loaded with PDGF-BB and KGN is presented in Figure 8. The fed PDGF-BB amount in the BSA solution was set as 10ng per μg BSA. Blank MPs without any cargo load, referred to as BLANK-M MPs, were also prepared using the same protocol and used as an analog to BSA/KGN-M or PDGF/KGN-M MPs for subsequent characterization and evaluation of hydrogels. The shell layer of some MPs was built using a mixture of CH and RDB-CH at a CH/RDB-CH ratio of 3 for viewing their core-shell structure via fluorescence microscopy.

### 4.3. Characterization

MPs were viewed using a scanning electron microscope (SEM, Quanta 200, FEI, Eindhoven, The Netherlands). The mean size of MPs was calculated from the measured diameters of 200 randomly selected MPs in SEM images for each sample using analysis software (ImageJ). The RDB-labeled shell layer of MPs was viewed using a confocal laser scanning microscope (Leica TCS SP5, Leica Microsystems, Buffalo Grove, IL, USA).

Some MPs were cryogenically ground into powder using a freezer mill (SPEX 6750, Metuchen, NJ, USA). A portion of the powder was extracted in PBS at 37 °C for 6 h using a shaking table. In the case of BSA/KGN-M MPs, BSA content in the extract was determined using a Bradford protein assay kit (Beyotime Biotechnology, Shanghai, China). KGN in the powder was extracted with DMSO, and the amount of KGN in the extract was detected at 277.8 nm using an ultraviolet-visible (U-V) spectrophotometer (TU-1901, Beijing Purkinje General Instrument Co., Ltd., Beijing, China). As for PDGF/KGN-M MPs, they were also cryogenically ground into powder, and the powder was extracted two times in PBS at 37 °C for 2 h per extraction using a shaking table. The collected extract was detected with a PDGF-BB ELISA kit to determine the concentration of PDGF-BB. The content of BSA and KGN was determined using the same methods applied to the BSA/KGN-M MPs.

The encapsulation efficiency (EE) of MPs and drug load (DL) was calculated by the following equations:

EE(%) = [W_0_/W_1_] × 100% (2)

DL = W_0_/W (3)

where W_0_ is the drug weight loaded in MPs, W_1_ refers to the feed-in weight of the drug, and W denotes the weight of MPs.

### 4.4. Preparation of Composite Solutions

BSA/KGN-M MPs were used with CH, SF, genipin and TYR to prepare a series of composite solutions with varied proportions, as shown in Table 2. These solutions were first mixed with a given amount of GP solution at 4 °C, and then processed into gels by incubating the mixtures in a water bath at 37 °C. The prepared composite solutions and gels were subject to rheological and mechanical measurements as well as other examination. Similarly, composite solutions containing PDGF/KGN-M MPs were also produced following the same method, and the prepared gels with formulations illustrated in Table 3 were used for subsequent release assessments.

Gelation time for gels was estimated with a tube inverting method. In a typical process, the prepared composite solution was stirred in an ice/water bath for 5 min, and then an aliquot (1.0 mL) of the above prepared solutions was introduced into a vial with stirring while being added with a prescribed amount of GP. The vial was then incubated in a water bath at 37 °C and the flowability of the solution was examined by inverting the vial every 20 s. Gelation time was recorded starting from the time point for the vial incubation and ending at the moment when the solution stopped flowing.

### 4.5. Rheological Measurements

A rheometer (Kinexus Pro KNX2100, UK) was used for rheological measurements. Frequency sweeps for elastic modulus (G′) and viscous modulus (G′′) of samples were detected in a frequency range between 0.1 and 100 Hz at 37 °C and constant strain amplitude of 1%. In the case of the temperature-dependence sweep, G′ and G′′ were recorded in a range between 25 and 45 °C at a temperature elevating rate of 1 °C/min using liquid samples. The incipient gelation temperature (T_i_) of gels was determined from the intersection of G′ and G′′ in the temperature-sweep spectra.

### 4.6. Mechanical Tests

Unconfined compression tests were conducted on a testing machine (MACH-1, Biomomentum) using cylindrical gel samples (10 mm in diameter and 7–8 mm in height). Samples were compressed at a constant strain rate of 10%/min. The compressive modulus (E) of samples was calculated using the slope of a line fitted to their respective stress-strain curves over 2–5% strain. The average compressive strain at the break for gels was used to estimate the elasticity of gels.

### 4.7. In Vitro Release Assessments

A typical process for the release test was illustrated as follows: Cylindrical gel samples (diameter: 8 mm) were prepared by filling the selected composite solution (0.5 mL) into a cylindrical mold and incubated at 37 °C for gelling. The prepared samples were placed in different vials followed by addition of 3mL of PBS. The vials were shaken on a rotating table at 37 °C and 60 rpm. At predetermined time points, 0.5 mL of medium was withdrawn and the same volume of fresh buffer was replenished. The released amounts of PDGF-BB and KGN were detected using a PDGF-BB ELISA Kit for the former and U-V spectrophotometry for the latter. Release profiles for PDGF/KGN-M MPs were also detected in the same way and used for making comparisons.

### 4.8. Cell Culture

Articular cartilage was harvested from the keen joints of New Zealand white rabbits (four weeks old). The isolated chondrocytes were cultured in DMEM supplemented with 10% fetal bovine serum (FBS), and 1% penicillin/streptomycin in an atmosphere of 5% CO_2_ at 37 °C, with medium change every 3 days. The cells at passage 2 were used for cell seeding.

CH solutions and SF solutions were introduced into glass dishes to form a thin-layer and the dishes were exposed to UV light at 4 °C for 4 h to sterilize the solutions. Two kinds of solutions were mixed while being added with sterilized BLANK-M MPs, genipin or/and TYR to produce composite solutions with their compositional proportions as shown in Table 2. For cell seeding, an aliquot of the composite solution was homogeneously mixed with a given volume of chondrocyte-containing culture medium to produce mixtures with a cell density of 8 × 10^6^ cells/mL. These mixtures were used for the follow-up cell experiments.

Live/Dead staining assay was performed to determine the viability of seeded cells. In brief, each cell-containing mixture (100 μL) was placed in a confocal dish, and incubated at 37 °C for gelling, followed by culture in complete medium with medium replacement every 2 days. At predetermined time points, the medium was removed and the gels were cultured with serum-free medium containing calcein acetoxymethyl ester (10 μM) and propidium iodide (15 μM) in the dark for 30 min at 37 °C for staining. After washing with PBS, the gels were imaged using a confocal microscope (LSM 510 META, Zeiss, Shanghai, China).

The DNA content in cell-seeded gels was measured to assess cell proliferation. Cell-containing mixtures were plated onto 24-well dishes (200 μL/well) and incubated at 37 °C for gelling. The gels were then cultured with complete medium. At prescribed time points, the gels were crushed into powder in liquid nitrogen and the resulting powder was digested with a proteinase K solution. The collected supernatant for each sample was analyzed by a Quant-iT PicoGreen dsDNA kit (Invitrogen) following the manufacturer’s instructions.

The above prepared supernatants for different samples were also subject to GAG measurements using the method described in our previous study [52]. Type-II collagen amount in these supernatants was measured using a Collagen Type II BioAssay ELISA kit (US Biological) following the manufacturer′s protocol. The amount of total collagen in the cell-seeded gels was estimated by detecting the amount of hydroxyproline first and then converting the amount of hydroxyproline into the total collagen amount at a hydroxyproline-to-collagen ratio of 0.125:1 [52,53].

### 4.9. Bioactivity Assessment

Balb/c 3T3 cells were purchased from Sixin Biological Technology Inc. (Shanghai, China). Cells were expanded in DMEM supplemented with 10% FBS, 1% penicillin/ streptomycin at 37 °C in a 5% CO_2_ atmosphere. The expanded cells were resuspended in PBS for use in subsequent tests. The activity of the released PDGF-BB was assessed by examining the chemotactic effect of PDGF-BB on Balb/c 3T3 cells [35,36]. 24-well Transwell culture plates (pore size of filter: 8.0 μm) were used for performing chemotaxis assay. The medium (serum-free DMEM), containing a prescribed amount of released PDGF-BB, was added to the lower chamber and Balb/c 3T3 cells were seeded into the inserts (1.5 × 10^4^ cells per insert). After 4-h incubation, the inserts were taken out and non-migrated cells on the upper surface of the filter were removed using a rubber wiper, and the cells moving to the lower surface of the filter were counted. Free PDGF-BB was used as a positive control.

### 4.10. Statistical Analysis

Data were presented as mean ± standard deviation. The statistical difference between groups was determined using one-way ANOVA. Differences were considered to be statistically significant at a level of *p* < 0.05.

## 5. Conclusions

Core-shell microspheres with a thick shell layer were successfully produced via a newly developed emulsification method using alginate and chitosan as core and shell materials, respectively. These microspheres were able to load kartogenin and PDGF-BB with high encapsulation efficiency due to the designed composition and processing methods. The composite gels composed of CH, SF and blank microspheres being crosslinked with genipin or/and tyrosinase were thermo-sensitive at physiological temperature and pH with mechanically strong and elastic features. By replacing blank microspheres with kartogenin and PDGF-BB loaded microspheres while following the same formulation and cross-linking strategy, the obtained composite gels showed the ability to administer the release of PDGF-BB and kartogenin in an adjustable temporal separation manner and to effectively preserve the bioactivity of the released PDGF-BB. In particular, some composite gels were capable of controlling the release of PDGF-BB and kartogenin in an approximately linear manner for several weeks. These results suggest that the optimally achieved composite gels have potential to function as an endogenous repair material for cartilage tissue engineering.

## Figures and Tables

**Table 1:** Parameters for three kinds of core-shell microspheres.

Sample Name	EE for BSA (%)	DL for BSA (μg/mg)	EE for PDGF-BB (%)	DL for PDGF-BB (ng/mg)	EE for KGN (%)	DL for KGN (μg/mg)	Mean Size (μm)	Mean Thickness of Shell Layer (μm)
BLANK-M	−	−	−	−	−	−	13.1 ± 1.71	2.94 ± 0.19
BSA/KGN-M	85.7 ± 4.12	2.48 ± 0.16	−	−	87.4 ± 2.39	34.96 ± 1.38	12.7 ± 1.57	3.17 ± 0.16
PDGF/KGN-M	86.3 ± 3.86	2.44 ± 0.19	87.4 ± 1.81	25.89 ± 0.61	88.1 ± 2.63	35.24 ± 1.51	13.5 ± 1.62	3.06 ± 0.12

**Table 2:** Parameters for hydrogels embedded with blank core-shell microspheres ^(a)^.

Sample Name	CH (*w*/*v*%)	SF (*w*/*v*%)	Genipin (*w*/*v*%)	TYR (μL) ^(c)^	BLANK-M MPs (*w*/*v*%) ^(d)^	pH	Gelation Time at 37 °C (sec) ^(^^e^^)^
GL-1 ^(b)^	2.0	−	−	−	−	6.94 ± 0.06	580 ± 23
GL-2	2.0	1.0	0.05	−	1.0	7.03 ± 0.08	295 ± 19
GL-3	2.0	1.0	0.05	−	2.0	7.01 ± 0.07	260 ± 16
GL-4	2.0	1.0	−	20	1.0	7.09 ± 0.06	285 ± 25
GL-5	2.0	1.0	−	20	2.0	7.11 ± 0.09	255 ± 19
GL-6	2.0	1.0	0.05	20	1.0	7.07 ± 0.08	230 ± 11
GL-7	2.0	1.0	0.05	20	2.0	7.14 ± 0.09	205 ± 10

^(a)^ The full volume of solutions was 1 mL; the concentration of GP for all these gels was 5.6 (*w*/*v*%). ^(b)^ GL-1 was used as control. ^(c)^ Concentration of TYR: 10000 U/mL. ^(d)^ See Table 1 for parameters of BLANK-M MPs. ^(e)^ Gelation time was determined by using a vial tilting or inverting method (time interval: 20 s).

**Table 3:** Parameters for hydrogels containing PDGF/KGN-M microspheres ^(a)^.

Sample Name	CH (*w*/*v*%)	SF (*w/v%*)	Genipin (*w/v%*)	TYR (μL) ^(b)^	PDGF/KGN-M MPs (*w/v%*) ^(c)^	PDGF-BB Content in Gel (ng/mL)	KGN Content in Gel (μg/mL)
GEL-I	2.0	1.0	0.05	−	1.0	258.9 ± 6.13	352.4 ± 15.13
GEL-II	2.0	1.0	0.05	−	2.0	517.8 ± 12.26	703.8 ± 30.26
GEL-III	2.0	1.0	−	20	1.0	258.9 ± 6.13	352.4 ± 15.13
GEL-IV	2.0	1.0	−	20	2.0	517.8 ± 12.26	703.8 ± 30.26
GEL-V	2.0	1.0	0.05	20	1.0	258.9 ± 6.13	352.4 ± 15.13
GEL-VI	2.0	1.0	0.05	20	2.0	517.8 ± 12.26	703.8 ± 30.26

^(a)^ The full volume of solutions was 1 mL; the concentration of GP for these gels was 5.6 (*w*/*v*%). ^(b)^ See Table 2 for the concentration of TYR solution. ^(c)^ See Table 1 for parameters of PDGF/KGN-M MPs.

**Table 4:** Kinetic parameters for microspheres and hydrogels.

Sample ^(^^a)^	*k*	*n*	*r* ^2^
PDGF/KGN-M (PGDE-BB)	50.11	0.28	0.9721
PDGF/KGN-M (KGN)	24.54	0.44	0.9784
GEL-II (PGDE-BB)	17.78	0.56	0.9816
GEL-I (PGDE-BB)	14.45	0.58	0.9892
GEL-II (KGN)	7.17	0.68	0.9828
GEL-I (KGN)	4.51	0.76	0.9840
GEL-IV (PGDE-BB)	19.95	0.55	0.9860
GEL-III (PGDE-BB)	15.84	0.55	0.9943
GEL-IV (KGN)	9.01	0.64	0.9847
GEL-III (KGN)	6.54	0.69	0.9869
GEL-VI (PGDE-BB)	7.94	0.71	0.9816
GEL-V (PGDE-BB)	4.98	0.79	0.9897
GEL-VI (KGN)	2.18	0.95	0.9763
GEL-V (KGN)	1.46	0.99	0.9703

^(a)^ See Table 1 and Table 3 for the definition of sample names.

## Data Availability

Not applicable.

## Supplementary Materials

The following supporting information can be downloaded at: https://www.mdpi.com/article/10.3390/biomimetics7020041/s1. Figure S1. Photos for sol-gel transition of several composite solutions that have their compositions corresponding to GL-1 (A), GL-3 (B), GL-5 (C) and GL-7 (D) in order; Figure S2. Number of migrating Balb/c 3T3 cells in response to PDGF-BB stimulation.
