# Supplementary material for: Strong and Elastic Chitosan/Silk Fibroin Hydrogels Incorporated with Growth-Factor-Loaded Microspheres for Cartilage Tissue Engineering
Source: Biomimetics (Basel). 2022 Apr 7;7(2):41. doi: 10.3390/biomimetics7020041 (PMC9036308; doi:10.3390/biomimetics7020041)
Supplement: Supplementary file 1 [file biomimetics-07-00041-s001.zip › biomimetics-1632277-supplementary.pdf]

Supporting information

Strong and elastic chitosan/silk fibroin hydrogels incorporated with growth factor-loaded microspheres for cartilage tissue engineering

Qing Min[1,†], Danlei Tian [2,†], Yuchen Zhang[1], Congcong Wang[2], Ying Wan[2,*], Jiliang Wu[1,*]

[1] School of Pharmacy, Hubei University of Science and Technology, Xianning 437100, P. R. China
[2] College of Life Science and Technology, Huazhong University of Science and Technology, Wuhan 430074, P. R. China

[†] These authors contributed equally to this work.

Dr. Ying Wan
ying_wan@hust.edu.cn

Dr. Jiliang Wu
E-mail: jlwu@hbust.edu.cn

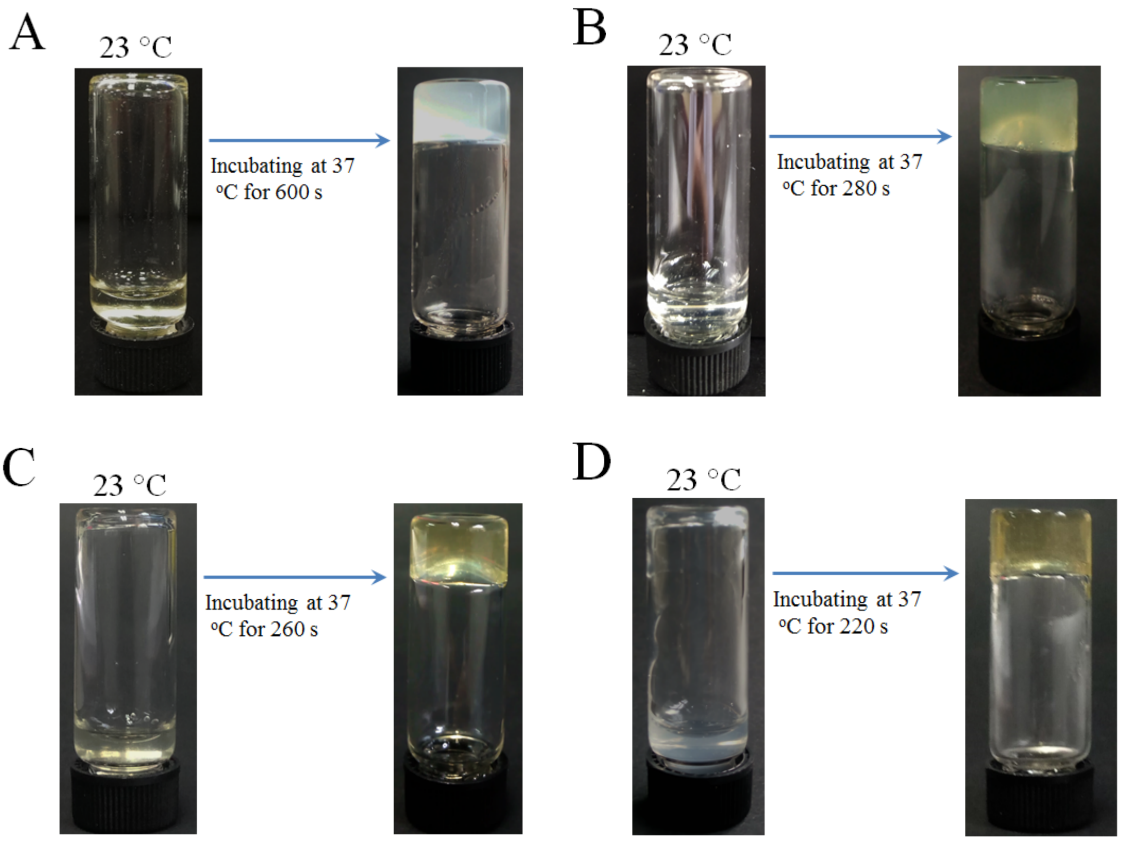

Figure S1. Photos for sol-gel transition of several composite solutions that have their compositions corresponding to GL-1 (A), GL-3 (B), GL-5 (C) and GL-7 (D) in order (see Table 2 for their parameters).

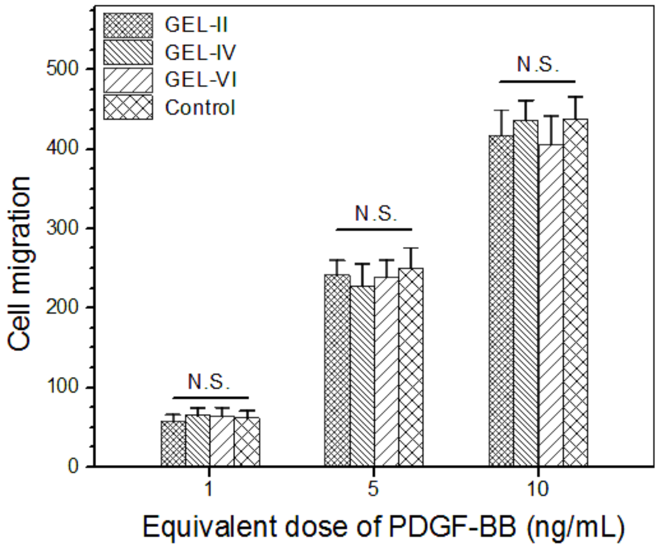

Figure S2. Number of migrating Balb/c 3T3 cells in response to PDGF-BB stimulation (culture time: 4 h; control: free PDGF-BB; N.S.: no significance).
